# Forkhead box N1 is possibly a novel biomarker and prognostic indicator for patients with squamous cell lung carcinoma

**DOI:** 10.7150/ijms.104616

**Published:** 2024-11-11

**Authors:** Xi Wang, Caihua Zhang, Bao-Jun Duan, Jun Bai, Tian Li, Xu-Yuan Dong, En-Xiao Li

**Affiliations:** 1 Department of Medical Oncology, The First Affiliated Hospital of Xi'an Jiaotong University, Xi'an, 710061, China; 2 Department of Medical Oncology, Shaanxi Provincial People's Hospital, Xi'an,710061, China; 3 Department of Oncology, People's Hospital Affiliated to Chongqing Three Gorges Medical College, Chongqing 404100, China; 4 School of Basic Medicine, Fourth Military Medical University, Xi'an, 710032, China

**Keywords:** Lung squamous cell cancer, FOXN1, Immunohistochemistry, Clinical characteristics, Prognosis, Biomarker

## Abstract

**Background:** The roles of Forkhead box N1 (FOXN1) in lung squamous cell carcinoma (LUSC) remains elusive. This study was focused on assessing the expression levels of FOXN1 in LUSC and exploring its potential clinical implications.

**Methods:** Utilizing a range of databases, this study conducted an analysis of the FOXN1 gene's expression levels, comparing LUSC samples with those from normal lung tissues. The expression levels of FOXN1 in primary LUSC and corresponding normal lung tissues were assessed using immunohistochemistry (IHC). Histoscore was used to evaluate the staining degree. χ2 test and Fisher's exact test were employed to assess the association between categorical variables that do not possess an ordinal nature. Multivariate survival analysis was conducted using the Kaplan-Meier method, the Wilcoxon test, and the Cox proportional hazards model.

**Results:** In contrast to normal lung tissues, the expression of the FOXN1 gene was found to be significantly elevated in LUSC tissues (*P* < 0.01). And FOXN1 was expressed in 79 (98.8%) evaluated LUSC tissues, most of which showed compositive IHC-staining intensity, presenting heterogeneously expression. 69 (87.3%) cases were characterized for strong immunostaining intensity, 70 (87.5%) cases showed moderate intensity, and 66 (82.5%) cases presented weak intensity. Only one sample of normal lung tissue, which represents 10% of the total, exhibited weak immunostaining exclusively (*P* < 0.05). Additionally, the expression of FOXN1 was found to have a significant correlation with the grading of LUSC, the presence of lymph node and distant metastases, the stage of the disease, and the survival outcomes (*P* < 0.05).

**Conclusion:** The expression of FOXN1 is frequently increased in LUSC, and the patients with high FOXN1 expression have a poorer survival outcome. FOXN1 can be a novel biomarker and prognostic indicator for LUSC patients.

## 1. Introduction

The 2022 Global Cancer Statistics report indicates that lung cancer continues to be the most prevalent type of cancer and remains the primary cause of cancer-related mortality worldwide 1-6. Non-small cell lung cancer (NSCLC) constitutes 85% of all lung cancer cases, with lung squamous cell carcinoma (LUSC) and lung adenocarcinoma (LUAD) being the predominant subtypes. LUSC accounts for nearly 30% of all NSCLC 7, the high mortality rate is attributed to the fact that many patients with LUSC are typically diagnosed at later stages, often with distant metastasis, which precludes them from being candidates for curative surgery 7. Besides, unlike LUAD, patients with LUSC lack specific treatments and they have not benefitted from targeted therapies, aggravating its poor prognosis. The 5-year survival rate for LUSC has been reported to be only 26% 8. Therefore, further research is required to identify possible biomarkers associated with LUSC in order to help develop diagnosis and treatment decision and guide individualized prognosis.

Low-dose computed tomography (LDCT) serves as an efficacious method for the early identification and screening of lung cancer. It is recognized for its potential to enhance patient prognosis and decrease lung cancer mortality rates, a practice that has been strongly endorsed by various clinical guidelines 9,10. Despite the technique's high sensitivity, it is crucial not to overlook the potential risks associated with the cumulative exposure to radiation and the possibility of overdiagnosis that can arise from the use of LDCT in lung cancer screening 11. Pathological and cytological examination remains the benchmark for diagnosing lung cancer 12. Extensive data have indicated that P40, P63, and cytokeratin 5/6 are specific biomarkers for the identification of LUSC, while Napsin A and transcription termination factor 1 (TTF1) were specific markers for LUAD. Therefore, TTF-1, P40, Napsin A and some other factors were recommended to differentiate LUAD and LUSC in multiple Guidelines for NSCLC 12. However, in certain instances, a widespread coexpression of TTF1 and p40 has been noted 13. Besides, over the past decades, numerous dysregulated genes involved in NSCLC have been identified as the potential biomarkers for prognosis, such as Glypican 3 (GPC3), Claudin-3 (CLDN3) and astrocyte-elevated gene-1 (AEG-1) 14. But each factor has its limitations and there are still no identified exact potential biomarkers for LUSC prognosis.

FOX family comprises a variety group of “winged-helix” transcription factors (TFs), has been confirmed to exert significant influence on the genesis and progression of tumors, holding the promise of serving as potential biomarkers for cancer 15-18, such as FOXO1, FOXP3, FOXM1, they have been confirmed to be involved in multiple signaling pathways in lung cancer and revealing its diagnostic and therapeutic potentials in NSCLC 17-19. As a constituent of the FOX family, FOXN1 has been documented in various types of tumors 20-22. but only a few studies investigated the expression of FOXN1 and its clinical significance. Some literatures reported FOXN1 acted as an oncogene, while others reported it may be a tumor suppressor gene 21,22. For example, Ghezzo MN *et al.* explored the role of FOXN1 in thymic leukemogenesis using a transgenic mouse model of T-cell acute lymphoblastic leukemia (T-ALL). Researchers found that FOXN1 haploinsufficiency delayed T-ALL onset and reduced leukemic cell expansion, highlighting its importance in thymic stromal cell regulation 21. While Ji X *et al.* investigates FOXN1 in NSCLC, revealing that FOXN1 acts as a tumor suppressor, especially in lung adenocarcinoma. Overexpression of FOXN1 inhibited cell proliferation and invasion, while its reduced expression promoted tumor growth. FOXN1 represses oncogenes EZH2 and β-catenin, linking it to better NSCLC prognosis 22. This suggests that we should contemplate the pivotal function and the clinicopathological traits of FOXN1 in the context of lung cancer. This research was conceived to appraise the expression levels of FOXN1 and the correlation between FOXN1 and clinicopathological traits in LUSC, and then could be executed as an element of the preliminary phase of a prefeasibility study for subsequent in-depth investigation.

## 2. Methods

### 2.1. Data extraction from multiple databases

The expression of the *FOXN1* gene in both lung squamous cell carcinoma (LUSC) and normal lung tissue was scrutinized through multiple databases, including Xena and TNMplot.com 23,24, which were feasible to do online analysis of multiple databases including Gene Expression Omnibus (GEO), Genotype-Tissue Expression (GTEx) and The Cancer Genome Atlas (TCGA). In addition to the aforementioned databases, the KM plotter was utilized to evaluate the influence of *FOXN1* expression on the survival rates of patients with LUSC. The KM plotter is a valuable online tool that amalgamates data from various repositories, including European Genome-phenome Archive (EGA), TCGA and GEO, to provide a Kaplan-Meier survival curve analysis. This analysis is pivotal for determining the prognostic significance of gene expression in cancer patients.

### 2.2. Sample collection

From 2021 to 2023, the normal lung tissues and primary LUSC samples were collected at the department of Pathology, the First Affiliated Hospital of Xi 'an Jiaotong University (Xi'an, Shaanxi Province, China). The informed consent of the patient was obtained for each specimen and clinical data. Ethic approval was granted by the ethics committee of The First Affiliated Hospital of Xi'an Jiaotong University (No. 2021-156). The patient's death date and subsequent information for living patients was procured through telephone monitoring and hospital records. All tissue specimens were subjected to macroscopic sectioning and microscopic histological examination performed by certified pathologists. The grading and diagnosis of the primary LUSC adhered to the criteria outlined by the World Health Organization (WHO). Subsequently, according to the American Joint Committee on Cancer (AJCC) manual, we determined the stage of tumor. Furthermore, a segment of the primary LUSC specimen was composed of tissue microarrays that were procured from Shanghai Zhuohao Medical Technology Limited Company (Shanghai, China) (ZL-LUC1602).

### 2.3. Immunohistochemistry and scoring of FOXN1 staining

Immunohistochemical (IHC) analysis was conducted on tissue sections derived from samples that were fixed with 4% buffered formalin and incorporated into paraffin, and then the paraffin-embedded tissue parts were deparaffinized and subjected to hematoxylin-eosin (HE) staining. a standard procedure for highlighting cellular structures in histological examination. Immunohistochemical staining was performed with anti-FOXN1 antibody (Rabbit polyclonal FOXN1, bs-6970R; Bioss, Beijing, China) with 1:150 fluxing, utilizing the Leica Bond Polymer Refining Assay Kit on the Leica Bond-max automatic dyeing system (Wetzlar, Germany).

The immunohistochemical (IHC) score for the tissues was executed through the utilization of a pathological semi-quantitative tissue score, which was also called histoscore (H-score). This scoring system is designed to amalgamate the intensity of the immunostaining, henceforth denoted as the IHC-score, with the proportion of tumor cells in the tissue section that demonstrate positive immunoexpression. The IHC score of FOXN1 was determined according to the intensity level of its nuclear staining. Tissue evaluation was categorized based on the IHC-score into four distinct grades: 0, indicating the absence of staining; 1+, signifying faintly perceptible nuclear staining in some areas; 2+, reflecting a moderate degree of nuclear staining; and 3+, denoting the presence of intense nuclear staining throughout histologic slice. In a word, H-score was calculated using the formula: H-score=Σ (pi × i), where pi represented the number of positive cells as a percentage of all cells in the tissue section and i represented the intensity of staining. Consequently, H-score scale expanded from 0, indicating that a portion of the tissue was completely negative and not stained, to a highest score with 300 points, representing this specimen with full 3+ staining across all cells. This scoring system more markedly distinguishes samples exhibiting high staining intensities and those presented primarily low staining intensities. In this study, all tissue samples underwent evaluation by two credentialed pathologists who operated in an independent manner. This approach ensures the reliability and objectivity of the histopathological assessments.

To determine the relationship between the level of FOXN1 expression and the clinicopathological features of patients who suffered from lung squamous cell carcinoma (LUSC), the samples were categorized into two distinct groups according to the median H-score: one group representing positive/high expression (≥ median) and the other representing negative/low expression (< median).

### 2.4. Evaluation of heterogeneous expression

Given the absence of established guidelines for evaluating heterogeneity among LUSC patients, this study referred to several scholarly articles. Based on the IHC-scores, we categorized heterogeneity into various groups to account for the variability observed in FOXN1 expression. If IHC-score 3+ and 0 appeared simultaneously and held more than 50% of the tumor tissue combined, it was regarded as the strong heterogeneous expression 25. Furthermore, the immunostaining patterns of these heterogeneous tumors were scrutinized with enhanced rigor. In LUSC, a subset of tumor cells exhibited a dispersed pattern characterized by minimal or absent IHC staining, a condition we have designated as “scattered”. Certain tumor samples demonstrated a notable reduction in immunostaining intensity with increasing depth from the tumor periphery, a phenomenon we have termed the "downward gradient" pattern. An additional form of tumor heterogeneity, characterized by a “patchy” distribution, was observed where a substantial concentration of tumor cells, distinctly clustered and with clearly defined boundaries, showed minimal or no immunostaining.

### 2.5. Statistical analysis

SPSS version 22.0 (IBM Corp., Armonk, NY, United States) was applied for statistical analysis. We used the χ2 and Fisher's exact test to evaluate the correlation between non-ordinal variables, and the The Benjamini-Hochberg method was employed to adjust for the false discovery rate (FDR) in the correlation analyses, ensuring the accuracy of the statistical inferences drawn. Furthermore, a multivariate analysis was conducted to determine if any significant factor associated with FOXN1 expression stood as an independent predictor. The median survival estimates were ascertained using the Kaplan-Meier method, complemented by the 95% confidence intervals (CIs). The Wilcoxon test was subsequently applied to evaluate disparities in median survival times. Moreover, the Cox proportional hazards model was engaged for a multivariate survival analysis to identify factors that significantly influence survival outcomes. *P* < 0.05 was a statistically significant threshold for the difference.

## 3. Results

### 3.1. Bioanalysis of gene expression of FOXN1 in LUSC from databases

**Figure 1:**
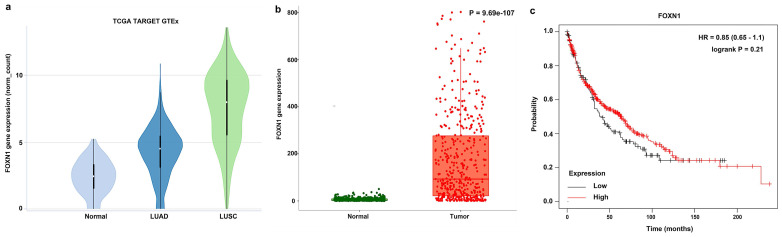
** Online analysis of gene expression of *FOXN1* and its effect on survival in lung squamous cell carcinoma (LUSC) using the database. (a)** Analysis of *FOXN1* expression in normal lung tissue and LUSC using Xena (http://xena.ucsc.edu/compare-tissue/) based on The Cancer Genome Atlas (TCGA) and Genotype-Tissue Expression (GTEx) databases; **(b)** Analysis of *FOXN1* expression in normal lung tissue and LUSC using the TNMplot.com (https://tnmplot.com/analysis/) based on TCGA, GTEx, and Gene Expression Omnibus (GEO) databases; **(c)** Assessment of the *FOXN1* effect on survival in LUSC using KM plotter (https://kmplot.com/analysis/) based on GEO, European Genome-phenome Archive (EGA), and TCGA databases. HR: Hazard ratio.

In the Xena analysis, a total of 288 normal tissue samples were compared alongside 574 LUAD and 548 LUSC samples. Our findings indicated that the gene expression levels of FOXN1 in LUSC were elevated significantly in comparison to both LUAD cases and normal lung tissues, with the discrepancy being statistically pronounced (*P* = 0.000) (Fig. 1 a). The gene expression analysis of FOXN1 conducted on TNMplot.com, which included 476 samples of normal lung tissues and 501 samples of LUSC, yielded results that corroborated the findings from the Xena analysis (Fig. 1 b). The KM plotter was applied to scrutinize the impact of FOXN1 expression on the survival of 524 patients with LUSC (Fig. 1 c).

### 3.2. FOXN1 expression in normal lung tissue and in primary LUSC

**Figure 2:**
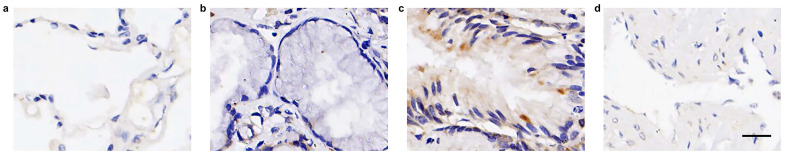
** Expression of FOXN1 in normal lung tissue. (a)** FOXN1-specific staining was not detectable in any of the normal pulmonary alveolar cells; **(b)** FOXN1-specific staining was not detectable in any of the normal bronchial submucosal glands; **(c)** FOXN1-specific staining was not detectable in any of the normal bronchus epithelial cells, but a little nonspecific staining was detectable; **(d)** FOXN1-specific staining was not detectable in any of the pulmonary artery. Scale bar 50μm.

In our examination of a collection of normal lung tissue samples (*n* = 10) for FOXN1 expression, it was observed that nine out of ten samples exhibited an absence of FOXN1-specific staining across all histological cell types and the various distinct structures of the normal lung tissue, including the pulmonary alveoli, bronchus, bronchial submucosal glands, and pulmonary artery. A single case displayed a faint nuclear staining for FOXN1 (Table 1). Representative images illustrating these findings are presented in Fig. 2.

Additionally, a total of 80 cases of primary LUSC were evaluated for FOXN1 expression. The patients' mean age was 61.6 years. Men make up the majority, and most of the LUSC samples were classified as poorly differentiated, that is, grade 3. Among these, 33 cases (41.2%) were staged as pT3/4, indicating a more advanced primary tumor. Furthermore, lymph node invasion (pN1/2/3) was confirmed in 37 cases (46.2%), and 17 cases (21.2%) had evidence of distant metastasis at the initial point of diagnosis. A summary of these demographic and clinical characteristics is provided in Table 2.

**Figure 3:**
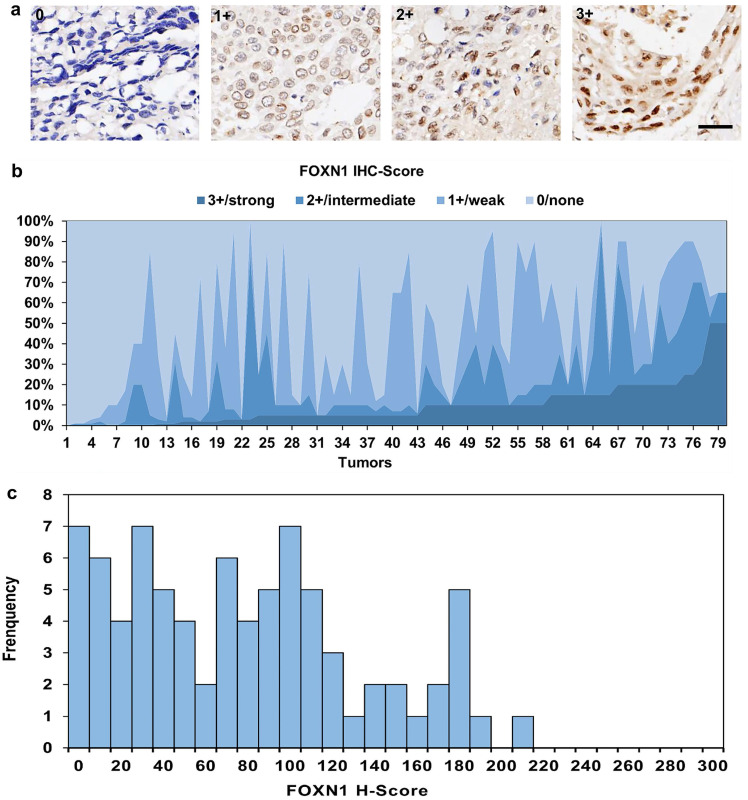
** Expression of FOXN1 in lung squamous cell carcinoma (LUSC). (a)** Examples of FOXN1-positive LUSC tissues with 0/none, 1+/weak, 2+/intermediate, and 3+/ strong staining intensity. Scale bar 50μm; **(b)** The IHC-score distribution of FOXN1 in 80 LUSC tissues; **(c)** Distribution and frequency of the Histoscore (H-Score) distribution in this study.

In contrast to normal lung tissues, FOXN1 displayed high expression rate in LUSC patients (*P* = 0.000), with 79 (98.8%) LUSC cases revealed positive expression (Table 1), in which most patients presented compositive IHC-staining intensity. 69 (87.3%) cases were scored up to 3+ IHC staining intensities, 70 (87.5%) cases were scored IHC 2+, 66 (82.5%) cases were scored IHC 1+ (representative pictures are showed in Fig. 3 a). The maximum expression of FOXN1 IHC 3+ was observed in 3 cases with 50.0% proportion of tumor cells. The IHC-score distribution of FOXN1 is exhibited in Fig. 3 b. Additionally, we compiled a summary of the distribution and frequency of the H-scores within our study. The range of H-scores spanned from 0 to a highest of 210. The median H-score for tumors that tested positive was recorded as 77 (Fig. 3 c).

### 3.3. FOXN1 correlates with distant metastasis, lymph node metastasis, grading, stage and survival

Comparative analysis between different groups revealed that FOXN1 expression is correlated with distant metastasis, lymph node metastasis, grading, and stage of LUSC (Table 2). In this study, the distant metastasis was evaluated in 80 cases, including M0 (*n* = 63) and M1 (*n* = 17). The expression levels of FOXN1 were observed to be significantly elevated in LUSC patients exhibiting distant metastases when contrasted with those at the M0 stage (76.5% *vs* 46.0%, *P* = 0.031). Additionally, our analysis revealed that 32 cases characterized by poor differentiation (grade G3) exhibited positive FOXN1 expression. In contrast, patients with well to moderately differentiated tumors (grades G1/G2) displayed significantly lower rates of FOXN1 expression, with a marked difference observed between the two groups (64.0% *vs* 33.3%, *P* = 0.011).

Moreover, in the process of analysis, we observed some interesting phenomena. The proportion of positive FOXN1 expression did not vary significantly across the different N categories (N0, N1, N2, and N3) when initially assessed. However, upon performing a stratified analysis, a correlation between FOXN1 expression and nodal involvement emerged, indicating that a more nuanced examination is necessary to discern the relationship between lymph node status and FOXN1 expression levels in LUSC patients. The cases with lymph node invasion (N1+N2+N3) showed significantly higher FOXN1 expression than the cases without lymph node invasion (64.9% *vs* 41.9%, *P* = 0.046). A similar outcome was also observed in stage group, in which FOXN1 positivity showed the following distribution: 7 (35.0%) cases in stage I; 11 (61.1%) cases in stage II; 11 (44.0%) cases in stage III; 13 (76.5%) cases in stage IV. Initially, no significant statistical difference in FOXN1 expression was observed between the various tumor stages. However, upon stratification of the tumor stages, a notable difference was identified. The expression of FOXN1 was found to be significantly higher in cases classified as stage IV compared to those at stages I, II, and III combined (76.5% for stage IV versus 46.0% for I+II+III, with a *P*-value of 0.031).

To determine if any significant factors associated with FOXN1 expression were independently linked, a multivariate analysis was conducted. Our investigation revealed that several significant factors—namely, distant metastasis, lymph node metastasis, tumor grading, and stage—were correlated with FOXN1 expression in an independent manner. For all these factors, the associated P values were below the threshold of 0.05, indicating a statistically significant relationship with FOXN1 expression levels.

We observed that no significant correlation was existed between the FOXN1 expression levels and the T category. No other clinicopathological characteristic of LUSC patients, such as age, gender, tumor site, or smoking status, correlated with FOXN1 expression (Table 2).

**Figure 4:**
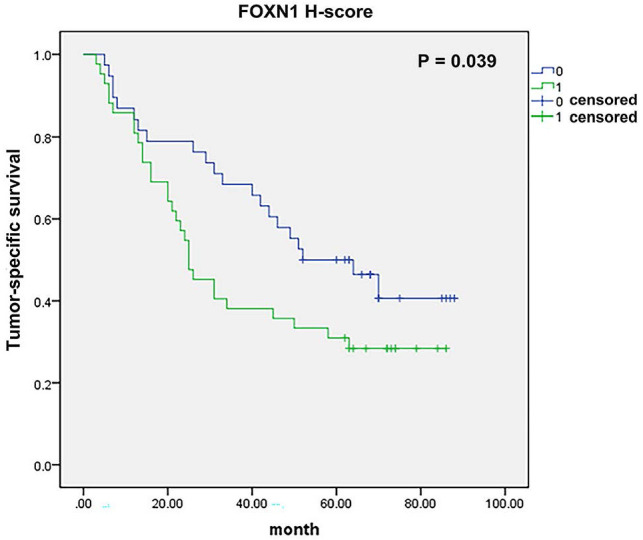
** FOXN1 and survival.** There was significant correlation between tumor-specific survival and FOXN1 expression in lung squamous cell carcinoma (38 *vs* 42 patients; median survival 52 months *vs* 25 months; *P* = 0.039).

Furthermore, tumor-specific survival data were accessible for a cohort of 80 patients, and our analysis revealed a meaningful correlation between the expression of FOXN1 and the cancer-specific survival rates in LUSC patients (Fig. 4, 38 vs 42 patients; median survival 52 months vs 25 months; *P* = 0.039). Besides, we have conducted additional multivariate analyses to account for potential confounding factors such as age, gender, and smoking status. The result shows FOXN1 expression remains a significant predictor of survival when controlling for these confounders.

### 3.4. FOXN1 is frequently heterogeneously expressed in LUSC

**Figure 5:**
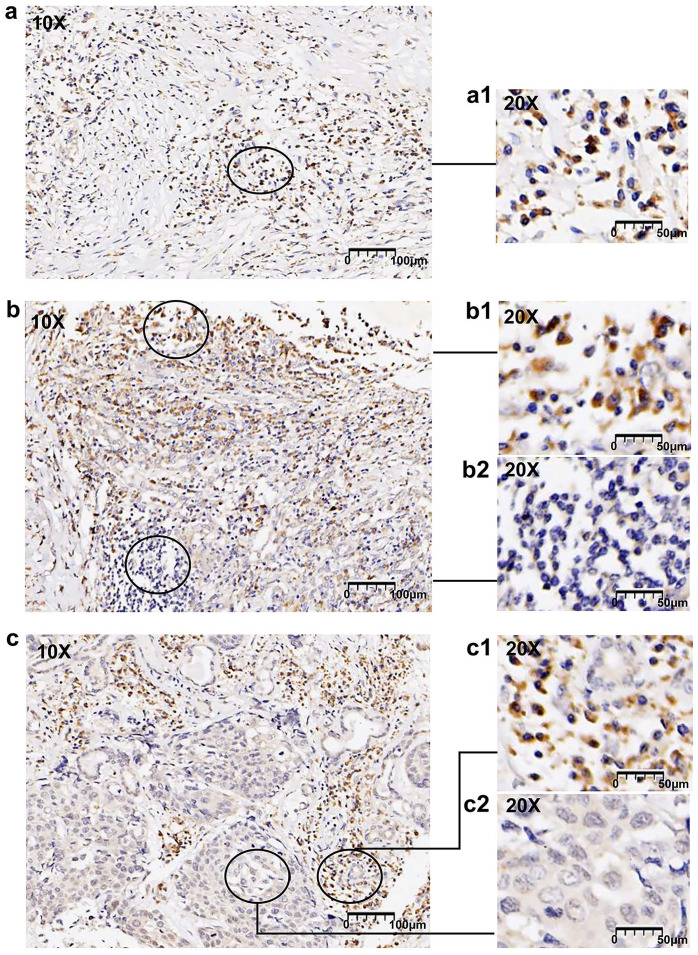
** FOXN1 heterogeneity patterns. (a)** Representative image of scattered pattern in lung squamous cell carcinoma (LUSC): randomly distributed cells with different immunostaining intensities, (a1) shows a 20x magnification; **(b)** Representative image of downward gradient in LUSC: declining immunostaining intensity towards the depth of the tumor tissue, (b1) and (b2) shows a 20x magnification; **(c)** randomly distributed and well demarcated areas of converged tumor cells with low or no staining, (c1) and (c2) shows a 20x magnification.

**Figure 6:**
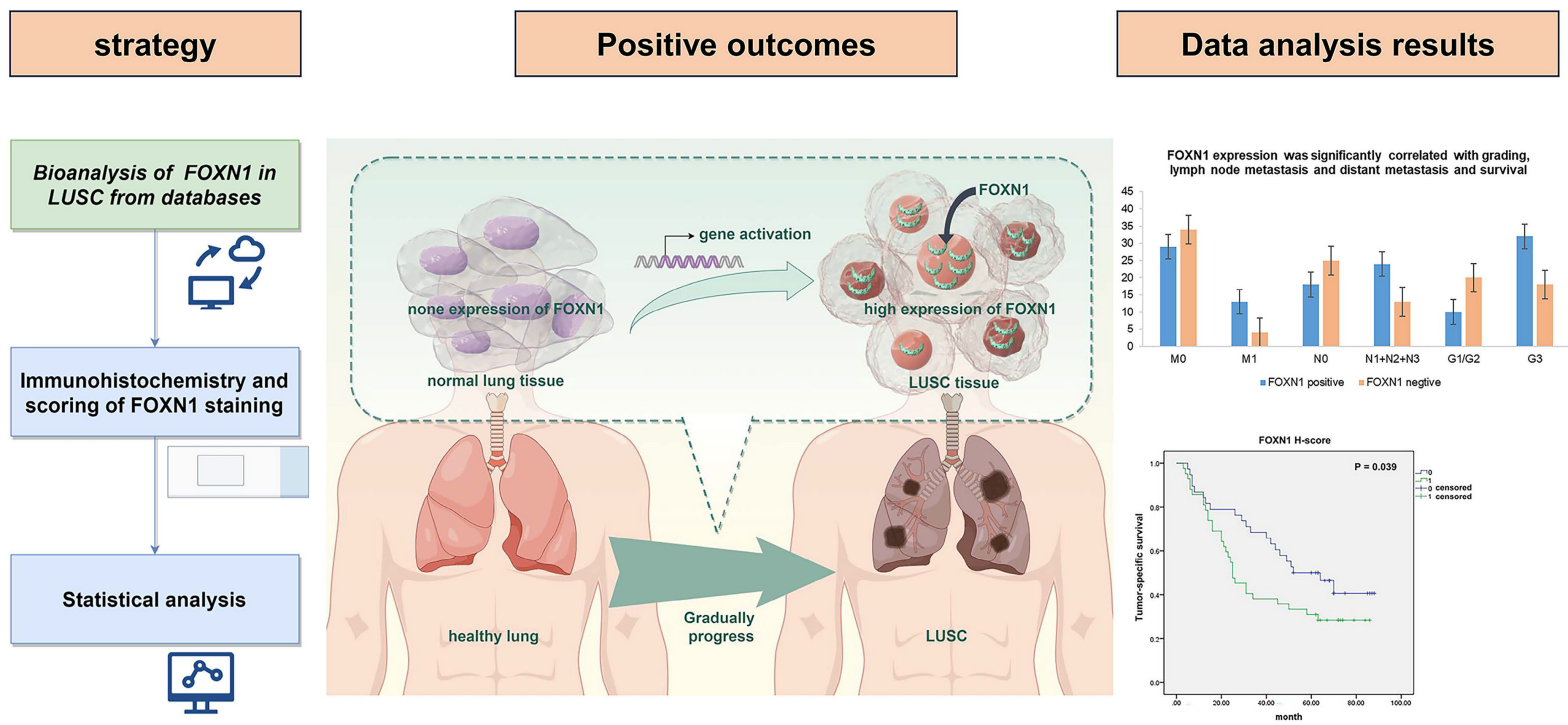
Graphical abstract of this study.

In this study, 38 (47.5% of 80) LUSC cases displayed compositive IHC-intensity, revealing the expression of FOXN1 demonstrated a pronounced tendency towards frequent heterogeneity within the tumor samples. We conducted an examination of the varying immunostaining distribution patterns observed among these heterogeneous cases. Among the 38 LUSC patients with heterogeneous expression, 20 (52.6%) exhibited a “scattered” pattern that presented a diffuse distribution of cancer cells with low or absent immunohistochemical (IHC) staining. Additionally, 10 (26.3%) of these patients displayed a “downward gradient” pattern, where the immunostaining intensity progressively diminished as the depth of the tumor tissue increased. The remaining 8 of 38 (21.1%) LUSC patients exhibited a “patchy” pattern. Representative images are displayed in Fig. 5. Graphic abstract is shown in Fig. 6.

## 4. Discussion

### 4.1. Main interpretation

Although there is a growing body of research on FOXN1, reports on its role in lung cancer are infrequent, and the specific mechanisms by which FOXN1 functions in LUSC remain largely unknown. Therefore, in this study, we conducted a bioanalysis of FOXN1 gene expression in LUSC using database resources and assessed FOXN1 expression in a substantial cohort of LUSC patients through IHC. By utilizing comprehensive datasets, including TCGA, GEO, GTEx and EGA, we were able to robustly analyze FOXN1 expression patterns and their prognostic implications in LUSC. This integration of multiple sources provides a more reliable and comprehensive understanding of FOXN1's potential role.

Additionally, the use of IHC on clinical samples further strengthens the study, offering direct validation of FOXN1 expression in LUSC tissues, complementing our bioinformatics findings. Subsequently, we examined its association with various clinicopathological features, including survival outcomes. To enhance the data's reliability and validity, we utilized the H-score method to scrutinize FOXN1 expression in LUSC, allowing for a clearer distinction of staining intensity among samples. Based on this approach, our study yielded several significant findings.

Firstly, our study confirmed a relatively high expression of FOXN1 in LUSC and much lower expression in normal lung tissues (Fig. 1 and Fig. 2). This differential expression of FOXN1 suggests that FOXN1 may be used as a potential diagnostic biomarker for LUSC and it could potentially serve as a valuable diagnostic instrument in routine surgical pathology practice. This has been also reported in some other cancer types. Nonaka D *et al.* documented that FOXN1 was consistently diffusely expressed in the nuclei of type B thymomas, but was typically found in a focal distribution within thymic carcinomas (76%) 26. This suggests that FOXN1 serves as a sensitive and specific marker for differentiating thymic carcinoma from thymoma. Ji X *et al.* showed that there was lower expression of FOXN1 in LUAD tissues and cell lines, which suggested us FOXN1 may be a biomarker to distinguish LUAD and LUSC 22. Besides, we recognize that FOXN1 could potentially serve as a biomarker for early-stage LUSC. Given its expression patterns, future studies could investigate whether FOXN1 plays a role in the early detection or screening of LUSC, particularly in high-risk populations. This line of research could be valuable for improving early diagnosis and treatment strategies. However, FOXN1 heterogeneity brings a great challenge to diagnostic evaluations in LUSC. Aligning with the distributions of IHC-score and H-score, this study uncovered a prevalent pattern of FOXN1 expression heterogeneity in LUSC (Fig. 5). We then delineated various types of heterogeneity, which may pose significant challenges to both scientific inquiry and clinical application. For instance, the “patchy” staining pattern could result in a significant misestimation of the overall expression rate when assessing a small tumor specimen. Additionally, the emergence of the “downward gradient” pattern, characterized by a marked decrease in immunostaining intensity with depth within the tumor tissue, might affect the reliability of deep tissue biopsies in LUSC. This is because conventional biopsies predominantly sample superficial tissues. Therefore, it is advisable to obtain as much tissue as possible during biopsy to enhance the diagnostic accuracy.

Secondly, the correlation analysis showed that the expression of FOXN1 was statistically higher in distant metastatic tumors, lymph node-positive tumors, grading G3 and stage IV LUSC patients. Distant metastasis and lymph node positivity are established independent prognostic factors associated with poor outcomes in NSCLC 27, so the high expression of FOXN1 may relate to poorer prognosis of LUSC patients. Additionally, by integrating bioinformatic analysis of FOXN1 gene expression with correlation studies, it has been observed that FOXN1 is typically silent in normal lung tissue yet becomes significantly upregulated during the onset and progression of malignant tumors, we thus assume that FOXN1 may act as an oncogene in the occurrence of LUSC and participate in the process of tumor differentiation and migration. Nevertheless, there is still very little research reported the role and molecular mechanism of FOXN1 in tumorigenesis, further experimentation is essential to validate this hypothesis and uncover the exact molecular mechanisms through which FOXN1 operates.

Thirdly, we found that FOXN1 expression is correlated with cancer survival of LUSC patients meaningfully, so it may be used as a potential prognostic biomarker for LUSC. But this was not in conformity to the result from the bioanalysis of various database (Fig. 1 c and Fig. 4). The reason for this discrepancy could be attributed to the difference in research focus: our study investigated the correlation between the expression of protein and patient's survival, whereas the database analysis evaluated the association between gene expression and survival in cancer. But this result is necessary to be verified in more LUSC patients. However, in general, comparison with well-known LUSC biomarkers, for example p63, p40, CK5/6, DSG3, C-MET, PD-L1, TP53, SOX2 and CDKN2A 12,28, which have been widely studied for their roles in LUSC diagnosis, progression, and prognosis. Our data suggest that high FOXN1 expression correlates with improved overall survival and might be involved in the metastasis of LUSC. Besides, it may be used as a marker for the diagnosis of LUSC. These founding positions FOXN1 as a complementary biomarker with both potential prognostic and diagnostic significance, especially in cases where conventional biomarkers may not fully predict outcomes.

Additionally, the vast majority of LUSC cases in this study showed a high positivity rate for FOXN1, while nearly all normal lung tissues showed FOXN1 negativity. Hence, FOXN1 may possess the potential to be developed as a therapeutic target. However, we should realize that FOXN1 is not a membrane protein, making it a difficult therapeutic target, and the high expression of a target does not necessarily represent optimize drug therapy 29,30. Fortunately, there are evidences suggest that FOXN1 expression may influence patient sensitivity to specific therapies, and it has potential role in guiding personalized treatment strategies. We explored potential links by drawing from the known biological roles of FOXN1, such as epithelial differentiation and immune regulation 31. These functions could have significant implications for treatment responses, particularly in immunotherapy and chemotherapy. For example, FOXN1's role in immune regulation raises the possibility that its expression may enhance the tumor's immunogenicity, making patients more responsive to immune checkpoint inhibitors, which have become a key therapy for LUSC. By correlating FOXN1 expression with treatment outcomes, FOXN1 could serve as a predictive biomarker, aiding in the selection of patients most likely to benefit from targeted therapies or immunotherapies. Future studies aimed at investigating these associations are crucial to establish the clinical utility of FOXN1 in personalized treatment planning.

### 4.2. Strengths and Limitations

This study utilized a well-defined cohort of LUSC patients and used both database analysis and immunohistochemistry, which provided a strong foundation for the conclusions drawn about FOXN1 expression. Besides, the significant correlation between FOXN1 expression and key clinical factors, including tumor grade, lymph node involvement, metastasis, and survival outcomes, underscores its potential as a prognostic indicator. Furthermore, the comprehensive analysis of FOXN1 expression in relation to clinical outcomes, which provides novel insights into its potential as a biomarker. But several limitations must be acknowledged.

Principally, the scope of our research was confined to the type of samples examined. We focused on the expression of FOXN1 in LUSC and did not extend our analysis to other subtypes of lung cancer. Secondly, we were confined by the quantity of samples and single institution. Thirdly, we acknowledge the absence of functional studies to validate the biological role of FOXN1 in LUSC. Looking ahead, it is essential that more rigorously designed and expansive studies be conducted to delve deeper into the expression patterns of FOXN1 in LUSC.

## 5. Conclusion

Based on the findings of this study, we noted a statistically significant increase in FOXN1 expression in LUSC tissues, which exhibited frequent heterogeneity in its expression patterns. Besides, a robust correlation was found between FOXN1 expression and various clinical features of LUSC, such as tumor grading, lymph node involvement, distant metastasis, disease stage, and patient survival. Patients exhibiting high levels of FOXN1 expression have a worse prognosis compared to the patients with low FOXN1 expression. Thus, FOXN1 is possibly a novel biomarker and prognostic indicator for LUSC patients.

In conclusion, our study describes a particular illustration for the expression of FOXN1 in LUSC and shows the possibility of FOXN1 act as a prognostic biomarker, and emphasizes the necessity for further research to translate these discoveries into clinical practice.

## Figures and Tables

**Table 1:** Classification of sample types investigated by FOXN1 staining.

Sample type	Samples, *n*	FOXN1 expression		*P* value
		Positive fraction ≥ 1%, *n* (%)	Staining intensity = 3+, *n* (%)	
LUSC	80	79 (98.8)	69 (87.3)	0.000*
Normal	10	1 (10.0)	0 (0.0)	

Notes: * *P* < 0.05; FOXN1, Forkhead box protein N1; LUSC, lung squamous cell carcinoma.

**Table 2:** FOXN1 expression and correlation with clinicopathological characteristics of Lung squamous cell carcinoma.

Clinicopathological parameter	Variable	Total valid,	FOXN1 expression		*P* value
		*n* (%)	Positive,* n* (%)	Negative,* n* (%)	
Age	< 62	33 (41.3)	17 (51.5)	16 (48.5)	1.000
	≥ 62	47 (58.7)	25 (53.2)	22 (46.8)	
Gender	Female	7 (8.8)	6 (85.7)	1 (14.3)	0.112
	Male	73 (91.2)	36 (49.3)	37 (50.7)	
Localization	Left	50 (62.5)	23 (46.0)	27 (54.0)	0.168
	Right	30 (37.5)	19 (63.3)	11 (36.7)	
smoking status	Smoking	13 (34.2)	7 (53.8)	6 (46.2)	1.000
	No Smoking	25 (65.8)	14 (56.0)	11 (44.0)	
T category	T1	13 (16.3)	8 (61.5)	5 (38.5)	0.631
	T2	34 (42.5)	15 (44.1)	19 (55.9)	
	T3	19 (23.7)	11 (57.9)	8 (42.1)	
	T4	14 (17.5)	8 (57.1)	6 (42.9)	
	T1 + T2+T3	66 (82.5)	34 (51.5)	32 (48.5)	0.774
	T4	14 (17.5)	8 (57.1)	6 (42.9)	
	T1 + T2	47 (58.8)	23 (48.9)	24 (51.1)	0.500
	T3 + T4	33 (41.2)	19 (57.6)	14 (42.4)	
	T1	13 (16.3)	8 (61.5)	5 (38.5)	0.554
	T2 + T3 + T4	67 (83.7)	34 (50.7)	33 (49.3)	
N category	N0	43 (53.8)	18 (41.9)	25 (58.1)	0.110
	N1	19 (23.7)	13 (68.4)	6 (31.6)	
	N2+N3	18 (22.5)	11 (61.1)	7 (38.9)	
	N0	43 (53.8)	18 (41.9)	25(58.1)	0.046*
	N1 + N2+N3	37 (46.2)	24 (64.9)	13 (35.1)	
M category	M0	63 (78.8)	29 (46.0)	34 (54.0)	0.031*
	M1	17 (21.2)	13 (76.5)	4 (23.5)	
AJCC stage	I	20 (25.0)	7 (35.0)	13 (65.0)	0.054
	II	18 (22.5)	11 (61.1)	7 (38.9)	
	III	25 (31.3)	11 (44.0)	14 (56.0)	
	IV	17 (21.2)	13 (76.5)	4 (23.5)	
	I	20 (25.0)	7 (35.0)	13 (65.0)	0.120
	II + III + IV	60 (75.0)	35 (58.3)	25 (41.7)	
	I + II	38 (47.5)	18 (47.4)	20 (52.6)	0.502
	III + IV	42 (52.5)	24 (57.1)	18 (42.9)	
	I + II + III	63 (78.8)	29 (46.0)	34 (54.0)	0.031*
	IV	17 (21.2)	13 (76.5)	4 (23.5)	
Grading	G1/G2	30 (37.5)	10 (33.3)	20 (66.7)	0.011*
	G3	50 (62.5)	32 (64.0)	18 (36.0)	

Notes: ^*^ *P* < 0.05; AJCC, American Joint Committee on Cancer; FOXN1, Forkhead box protein N1.

## Abbreviations

FOX: Forkhead box
LUSC: Lung squamous cell carcinoma
IHC: Immunohistochemistry
NSCLC: Non-small cell lung cancer
LUSC: Lung squamous cell carcinoma
LUAD: Lung adenocarcinoma
LDCT: Low-dose computed tomography
TTF1: transcription termination factor 1
GPC3: Glypican 3
CLDN3: Claudin-3
AEG-1: astrocyte-elevated gene-1
TFs: transcription factors
TCGA: The Cancer Genome Atlas
GEO: Gene Expression Omnibus
GTEx: Genotype-Tissue Expression
EGA: European Genome-phenome Archive
AJCC: American Joint Committee on Cancer staging system
FDR: false discovery rate
HE: hematoxylin and eosin
